# Prevalence, Clinical Factors and Impact of Dysphagia After Cardiac Surgery for Congenital Heart Disease

**DOI:** 10.1007/s00246-025-03953-y

**Published:** 2025-07-10

**Authors:** Sarah Hahn, Susan Willette, Amy Lay, James Schroeder, Inbal Hazkani, Taher Valika, Saied Ghadersohi

**Affiliations:** 1 Department of Speech Pathology, Ann and Robert H. Lurie Children’s Hospital of Chicago, Chicago, IL USA; 2 Department of Pediatric Cardiology, Ann and Robert H. Lurie Children’s Hospital of Chicago, Chicago, IL USA; 3 Division of Pediatric Otolaryngology – Head and Neck Surgery, Ann and Robert H. Lurie Children’s Hospital of Chicago, 225 East Chicago Ave, Box #25, Chicago, IL 60611 USA; 4 Department of Otolaryngology Head and Neck Surgery, Feinberg School of Medicine, Northwestern University, Chicago, IL USA

**Keywords:** Congenital heart disease, Cardiac surgery, Dysphagia, Aspiration, Vocal fold impairment, Pediatric feeding

## Abstract

Investigate the prevalence, instigating factors, and clinical impact of dysphagia in the perioperative period after cardiac surgery for congenital heart disease (CHD) to develop a management protocol. We evaluated patients that underwent selected cardiac surgeries for CHD that had a feeding evaluation over a 5-years period from 2018 to 2023 at a university based, tertiary care urban pediatric hospital. Demographic information, medical and surgical history, vocal fold mobility, clinical and instrumental swallow evaluation findings and feeding modality were reviewed. There were 398 (predominately infant) patients, median age at surgery was 1.1 (IQR 0.20–9.9) months with 181 (46%) females. A clinical and/or instrumental feeding evaluation was performed in all patients. Tube feeding was the primary means of feeding in 232 (58.3%) of patients at the time of initial postoperative discharge. Postoperative instrumental swallow data was available in 198 (49.7%) of patients. The median dysphagia outcomes and severity score (DOSS) of these patients was 4 (IQR2-5). Multivariable analysis demonstrated that single ventricle cardiac disease (OR 5.1, *p* = 0.018), vocal cord motion impairment (VFMI) (OR 5.6, *p* = 0.002), clinical concern for aspiration on postoperative evaluation (OR 6.1, *p* = 0.005), and a longer time between surgery and post-surgery clinical evaluation (OR 26.7, *p* = 0.048) were associated with need for tube feeding at discharge. Perioperative dysphagia requiring tube feeding is common after cardiac surgery for patients with CHD. The incidence is influenced by predicable factors. A protocolized approach utilizing these factors to screen for and manage perioperative dysphagia in these patients can improve their care.

## Introduction

Congenital heart disease (CHD) is the most common congenital abnormality, ranging from 8 to 9.5 per 1,000 live births globally [1, 2]. Children with CHD often have perioperative dysphagia, ranging from 18 to 83%, that can significantly impact their ability to gain weight, increase their need for supplemental nutrition/hydration, increase their length of hospital stay, and exacerbate caregiver burden and stress [3–5]. The definition of dysphagia and/or feeding disorders varies greatly [6]. According to Azer et al., dysphagia can be defined subjectively (by patients) as difficulty swallowing, and objectively (by clinicians) as an impairment in swallowing [7]. Severe dysphagia, in this study, was quantified by the need for tube feeding upon discharge and using the Dysphagia Outcome and Severity Score (DOSS) an objective measurement based on findings on a Video Fluoroscopic Swallow Study (VFSS) [8].

The presence and impact of perioperative dysphagia in children after congenital cardiac anomaly repair is well described [3–5]. Factors associated with the development of perioperative dysphagia in children after surgical repair include the underlying cardiac pathology, surgical approach for repair, and length of time on bypass. In addition, the presence of associated syndromes, respiratory instability in the postoperative period and postoperative chylothorax, vocal fold motion impairment (VFMI), and diaphragm paresis are all associated with an increased risk of dysphagia [3, 6]. There is no single well-established clinical assessment protocol for identifying and managing perioperative dysphagia in this at risk population [9]. Standardized protocols and multidisciplinary assessments are crucial to establishing and maintaining safe and effective feeding plans for these children. The goal is to optimize nutrition and adequately inform and support caregivers on the feeding and swallowing difficulties their children may face, as parents often report feeding difficulties as their greatest challenge [10–12].

Our study evaluates the factors that influence perioperative dysphagia in children undergoing cardiac surgery for CHD and proposes a standardized, multidisciplinary, postoperative feeding protocol. Risk factors identified in this cohort can help predict and manage those patients at risk of dysphagia requiring tube feeding at discharge. This will equip clinicians with the information necessary to manage perioperative feeding plans in these patients and to better prepare and support their families.

## Methods

Approval for this study was obtained from the Institutional Review Board at The Ann & Robert H. Lurie Children’s Hospital of Chicago (IRB# 2023–6082). This is a retrospective case series of children (mostly infants) that had undergone selected cardiac surgeries for congenital heart disease between January 1, 2019 and January 1, 2024. Using CPT codes an electronic medical record search was performed to identify patients who had undergone the following procedures: patent ductus arteriosus (PDA) ligation, Norwood procedure, Blalock-Thomas-Taussig shunt, bidirectional Glenn, Aortic Arch repair, coarctation of Aorta repair, aortic root replacement, vascular ring repair, arterial switch operation, Truncus arteriosus repair, heart transplant and left ventricular assist device (LVAD) placement. These procedures were chosen as they are often associated with dysphagia and/or VFMI, albeit through different mechanisms. In patients that had multiple surgeries, the surgery that was closest in date to the diagnosis of dysphagia or VFMI was considered the index surgery.

**Fig. 1:**
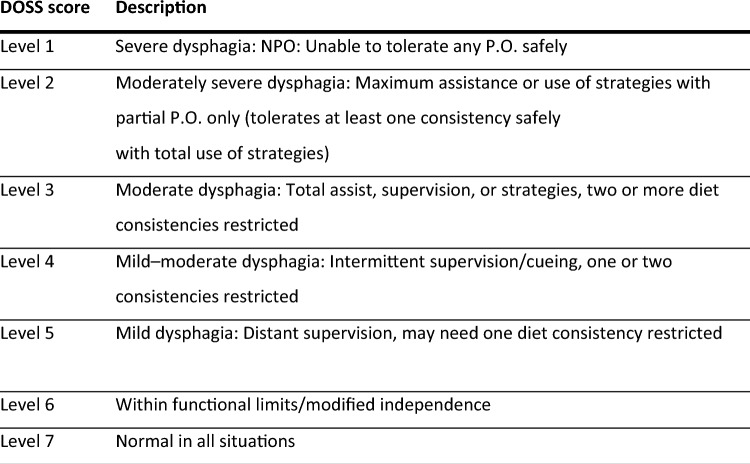
DOSS—Dysphagia outcome severity score

Patients were excluded if they did not have either a clinical or instrumental speech language pathologist (SLP) feeding evaluation. At the time of this manuscript, our institution's practice includes automatic SLP orders for surgical patients under 12 months of age and as needed orders for those over 12 months of age. Dysphagia in this study was operationally defined by the need for tube feeding (which could be due to dysphagia or nutritional requirements for adequate weight gain) and further qualified using the Dysphagia Outcome and Severity Score (DOSS), an objective measurement based on findings on a Video Fluoroscopic Swallow Study (VFSS) or fiberoptic endoscopic evaluation of swallowing (FEES). A modified numerical system ranging from 1 to 7 was created based on DOSS severity rating (Fig. 1). Thickened diet recommendations (International dysphagia diet standardization initiative (IDDSI) were also recorded [8, 13].

Demographic information, history of prematurity and history of single ventricle cardiac disease were noted. Comorbidities assessed included a history of extracorporeal membrane oxygenation (ECMO), genetic syndrome, neurologic comorbidity, airway abnormality, lower airway abnormality, tracheostomy and gastrostomy tube status. Surgical complications such as VFMI, chylothorax and diaphragm paresis/paralysis were also noted. The diagnosis of VFMI and diaphragm paresis was confirmed by fiberoptic laryngoscopy and imaging, based on postoperative clinical suspicion.

Demographic and clinical characteristics were reported as frequencies and percentages for categorical variables, means and standard deviations for continuous variables and median with interquartile range (IQR) for ordinal or parametrically distributed continuous variables. The patients were grouped based on the need for tube feeding versus those on a full PO diet (thin or thickened liquid) at initial discharge. The presenting symptoms and management were compared with chi squared testing, Wilcoxon rank sum test, and Student’s t-test where applicable. Multivariate logistic regressions were performed to assess variable effects on the need for tube feeding at discharge. Significance was determined at *p* < 0.05. All statistical analyses were performed using Stata 14.1 (Statacorp, College Station, TX).

## Results

### Cohort Description

There were 472 patients with CHD that had underwent cardiac surgery. Seventy-four patients were excluded as they did not have a clinical and/or instrumental swallowing evaluation leaving 398 patients in the study. The median age at surgery was 1.1 (IQR 0.20–9.9) months with 310 (77.9%) at less than 12 months of age and 198 (49.8%) at less than 1 month of age. There were 181 (45.5%) female patients. Common conditions in our cohort included a single ventricle cardiac diagnosis (*n* = 130, 32.9% and neurological comorbidity (*n* = 137, 34.4%). Most patients underwent palliative procedures for single ventricle disease or coarctation of the aorta/aortic arch repair (*n* = 202, 50.8%). Surgical complications included chylothorax (*n* = 75, 18.8%), and diaphragm paralysis (*n* = 24, 6.0%). Laryngoscopy was performed in 167 patients based on clinical suspicion from either postoperative voice changes or aspiration on instrumental swallow study and VFMI was found in 90 (53.9%). Table 1 summarizes the demographic, background conditions and postoperative complications of the patients.

**Table 1:** Demographics and baseline clinical data

	**All patients**
Number of patients (N)	398
Age at surgery (months)	Median 1.1 (IQR 0.20 to 9.9)
Female Gender	181 (45.5%)
Prematurity	34 (8.6%)
Single ventricle cardiac diagnosis	131 (32.9%)
Surgical procedure	
PDA ligation	12 (3.0%)
Norwood	41 (10.3%)
PDA stent/pulmonary banding	37 (9.3%)
BTT shunt	29 (7.3%)
Aortic root repair	2 (0.5%)
Coarctation of Aorta/Aortic arch repair	95 (23.9%)
Vascular ring repair	42 (10.6%)
Arterial switch operation	47 (11.8%)
Truncus arteriosus repair	9 (2.3%)
Heart transplant	50 (12.6%)
LVAD	28 (7.0%)
Other	6 (1.5%)
Comorbidities	
ECMO	20 (5.0%)
Syndromic	61 (15.3%)
Neurological	137 (34.4%)
Airway abnormality	44 (11.1%)
Lower airway abnormality	50 (12.6%)
Tracheostomy	41 (10.3%)
Gastrostomy tube	68 (17.1%)
Nissen	4 (1.0%)
Surgical complications	
VFMI	
Laryngoscopy performed	167 (42.0%)
VFMI after surgery	90 (53.9%)
Chylothorax	75 (18.8%)
Diaphragm paralysis	24 (6.0%)

*PDA* Patent ductus arteriosus, *BTT* Blalock-Thomas-Taussig, *LVAD* Left ventricular assist device, *ECMO* Extracorporeal membrane oxygenation, *VFMI* Vocal fold motion impairment

We subclassified the 398 patients based on tube feeding status at initial discharge after cardiac surgery. There were 166 (41.7%) patients that were discharged on a full PO diet of either thin or thickened liquids. The remaining 232 (58.3%) patients required tube feeding either as the primary diet or for supplemental nutrition in addition to oral feeding using thin or thickened liquids.

### Clinical Swallow Evaluation

Data from a preoperative clinical swallowing evaluation were available in 168 (42.2%) patients. Clinical concern for aspiration including findings of reduced airway protection, wet vocal quality or upper airway congestion was found in 51 (30.4%) patients (Table 2). Data from a postoperative clinical swallowing evaluation were available in 350 patients (87.9%). Clinical concern for aspiration was noted in 221 (63.4%) patients and 154 (74%) were discharged on tube feeding with a median DOSS of 3 (IQR2-5). Clinical signs of aspiration at both the preoperative clinical swallowing evaluation (p = 0.008 (OR 2.9 CI 1.2–7.4)) and postoperative clinical swallowing evaluation (*p* < 0.0005 (OR 3.2 (CI2.0–5.1)) were associated with need for tube feeding at initial discharge. In addition, the time between surgery and postoperative clinical swallow evaluation was found to be associated with need for tube feeding at discharge (*p* < 0.0005, Table 2).

**Table 2:** Clinical swallow evaluation data

		All patients	No tube feeding	Tube feeding	Univariate *p*-value
Preoperative clinical eval	N	168	54	114	
	Clinical concern for aspiration*	51 (30.4%)	9 (16.7%)	42 (36.8%)	**0.008**
Time between preclinical evaluation and surgery (months)		*n* = 158 Median 0.23 (IQR 0.1–0.73)	*N* = 50 Median 0.17 (IQR 0.07–0.67)	*N* = 108 Median 0.23 (IQR 0.13–0.82)	0.09
Postoperative clinical eval	*N*	350	142	208	
	Clinical concern for aspiration*	221 (63.4%)	67 (47.2%)	154 (74.0%)	** < 0.0005**
Time between surgery and postoperative evaluation (months)		*n* = 336 Median 0.13 (IQR 0.07–0.28)	*N* = 137 Median 0.07 (IQR 0.03–0.13)	*N* = 199 Median 0.2 (IQR 0.13–0.5)	** < 0.0005**

Significance was determined at *p*<0.05

^*^Clinical concern for aspiration included findings of reduced airway protection or wet vocal quality or upper airway congestion

### Instrumental Swallow Evaluation

Postoperative swallow study information was available in 198 (49.7%) patients as some patients were managed with clinical evaluation only (Table 3). The majority, 180 patients, had a VFSS while 18 had a FEES as their initial postoperative swallow assessment. Swallow studies were obtained a median 0.65 months (IQR 0.4–1.8) after the surgery date. The overall DOSS had a median of 4 (IQR2-5), the DOSS was worse in patients that required tube feeding at discharge indicating more severe dysphagia burden in this group (p < 0.00005). In the group of patients who required tube feeding at discharge, 133 (83.1%) had tube feeding as their primary diet (Median DOSS 3, IQR 2–4), while 27 (16.9%) used the tube for supplemental nutrition (Median DOSS 4, IQR 3–5). Ninety-seven (49%) of the patients who required both oral and tube feeding were using thickened liquids for PO feeding. Only 20 (12.5%) were discharged exclusively tube fed.

**Table 3:** Instrumental Swallow study data

	All patients	No tube feeding	Tube feeding	Univariate *p*-value
*N*	198	38	160	
Severity of dysphagia (DOSS)	Median 4 (IQR2-5)	Median 4.5 (IQR4-5)	Median 3 (IQR2-5)	** < 0.00005**
Surgery to initial swallow (months)	Median 0.65 (IQR 0.4–1.8)	Median 0.33 (IQR 0.2–2.1)	Median 0.65 (IQR 0.4–1.8)	0.05
Tube feeding primary diet	133 (67.2%)	0 (0%)	133 (83.1%)	
Tube feeding supplemental diet	27 (13.6%)	0 (0%)	27 (16.9%)	
Thickened diet	97 (49%)	14 (36.8%)	83 (51.3%)	
No PO diet	20 (10%)	0 (0%)	20 (12.5%)	

Significance was determined at *p*<0.05

*DOSS* Dysphagia outcome and severity score

### Factors Associated with Severe Dysphagia Requiring Tube Feeding

Univariate analysis and multivariate logistic regression analysis were used to assess factors associated with the need for tube feeding at initial discharge after cardiac surgery. Table 4 lists several factors that were statistically associated with tube feeding using univariate analysis. Multivariate logistic regression was performed to help control for these multiple-associated factors (Table 5). A stepwise procedure was used for multivariate model construction, with a *P*-value of 0.15 to enter the model and a *P*-value of 0.05 to remain in the model. This demonstrated that patients with a single ventricle cardiac diagnosis (OR 5.1, p = 0.018), vocal cord motion impairment (OR 5.6, p = 0.002), clinical concern for aspiration on postoperative evaluation (OR 6.1, *p* = 0.005), and a prolonged time between surgery and post-surgery clinical evaluation (OR 26.7, *p* = 0.048) were more likely to require tube feeding at discharge.

**Table 4:** Comparison of tube feeding vs no tube feeding patient groups

	No tube feeding	Tube feeding	Univariate p-value and OR (odds ratio)
*N*	166	232	
Age at surgery (months)	Median 5.2 (IQR 0.26 to 48)	Median 0.52 (IQR 0.20 to 4.5)	** < 0.00005**
Female Gender	70 (42.2%)	111 (47.8%)	0.262
Prematurity	9 (5.4%)	25 (10.8%)	0.06
Single ventricle Cardiac diagnosis	29(17.5%)	102 (43.9%)	** < 0.0005 (OR 3.7 CI 2.3–6.2)**
Surgical procedure			
PDA ligation	5 (3.0%)	7 (3.1%)	0.998
Norwood	3 (1.8%)	38 (16.8%)	** < 0.0005 (OR 10.6 CI 3.3–54.6)**
PDA stent/pulmonary banding	12 (7.2%)	25 (10.8%)	0.23
BTT shunt	7 (4.2%)	22 (9.5%)	**0.046 (OR 2.4 CI 0.95–6.7)**
Aortic root	1 (0.6%)	1 (0.43%)	0.812
Coarctation of Aorta/Aortic arch repair	40 (24.1%)	55 (23.7%)	0.928
Vascular ring repair	34 (20.5%)	8 (3.4%)	** < 0.0005 (OR 0.14 CI 0.05—0.32)**
Arterial switch operation	26 (15.6%)	21 (9.1%)	**0.044 (OR 0.54 CI 0.28–1.0)**
Truncus arteriosus repair	2 (1.2%)	7 (3.1%)	0.23
Heart transplant	25 (15.1%)	25 (10.8%)	0.204
LVAD	10 (6.0%)	18 (7.8%)	0.505
Other	1 (0.6%)	5 (2.2%)	0.21
Comorbidities			
ECMO	9 (5.4%)	11 (4.7%)	0.759
Syndromic	18 (10.8%)	43 (18.5%)	**0.036 (OR 1.9 CI 1.0–3.4)**
Neurological	33 (19.9%)	104 (44.8%)	** < 0.0005 (OR 3.3 CI 2.0–5.4)**
Airway abnormality	13 (7.8%)	31 (13.3%)	0.083
Lower airway abnormality	14 (8.4%)	36 (15.5%)	**0.036 (OR 2.0 CI 1.0–4.1)**
Tracheostomy	11 (6.6%)	30 (12.9%)	0.041
Nissen	1 (0.6%)	3 (1.3%)	0.496
Surgical complications			
VFMI			
Laryngoscopy performed	35 (21.1%)	132 (56.9%)	
VFMI after surgery	9 (5.4%)	81 (34.9%)	** < 0.0005 (OR 4.6 CI 1.9—12)**
Chylothorax	19 (11.4%)	56 (24.1%)	**0.001 (OR 2.5 CI 1.4- 4.6)**
Diaphragm paralysis	5 (3.0%)	19 (8.2%)	**0.032 (OR 2.0 CI 1.0–10)**

Significance was determined at *p*<0.05

*PDA* Patent ductus arteriosus, *BTT* Blalock-Thomas-Taussig, *LVAD* Left ventricular assist device, *ECMO* Extracorporeal membrane oxygenation, *VFMI* Vocal fold motion impairment

**Table 5:** Multivariable logistic regression models for factors associated with tube feeding at discharge

Variables	Odds ratio (95% confidence interval)	*P*-value	C-statistic/area under ROC
Single Ventricle cardiac diagnosis	OR 5.1 (1.3–19.3)	***p* = 0.018**	0.8576
Vocal cord motion impairment (VFMI)	OR 5.6 (1.8–17.1)	***p* = 0.002**	
Clinical concern for aspiration on postoperative evaluation	OR 6.1 (1.7–21.4)	***p* = 0.005**	
Time between surgery and postoperative evaluation (months)	OR 26.7 (1.0–689.1)	***p* = 0.048**	

Significance was determined at *p*<0.05

Multivariable logistic regression models associated with feeding tube need

A stepwise procedure was used for multivariable model construction, with a *P*-value of 0.15 to enter the model and a *P*-value of 0.05 to remain in the model

## Discussion

Feeding difficulties and dysphagia in children with CHD is common but rates are highly variable, ranging from 18 to 83% [3, 4]. Oral feeding difficulties are associated with a prolonged hospital stay (LOS) and an increased need for tube feeding at discharge [14]. Some form of tube feeding is required in 45%–75% of patients with CHD and/or single ventricle physiology upon discharge [15]. Approximately 58.3% of patients in our cohort were discharged with tube feeding, this rate was even higher (68.2%, *p* < 0.0005) in those that had surgery at less than 1 month of age. Optimal nutritional management, whether by oral or enteral routes, is key when caring for patients with CHD secondary to a demonstrated higher mortality rate associated with poor growth [16, 17]. Between 15 and 41% of infants born with CHD develop malnutrition and insufficient growth leading to delayed or complicated cardiac surgeries [18]. Multidisciplinary management targeting the identification of nutritional risk factors such as presence of dysphagia, associated with poor growth is essential in this at risk population.

The etiology of feeding difficulties and dysphagia are multifactorial, inconsistently defined and often identified without protocolized practices. Therefore, a universal or consistent method for and timing of feeding assessment in this population would be useful. These protocols would not only help identify dysphagia and need for tube feeding but also have significant impact on quality of life for caregivers and patients by providing valuable pre and postoperative education surrounding the risk factors for dysphagia and tube feeding necessity at time of discharge [6, 9, 19–21].

### Preoperative and Postoperative Clinical Swallowing Exam

Pre and post-operative feeding assessments are important tools used to better understand and support oral feeding skills.[22]; however, there is a lack of consensus in the literature regarding how pre or postoperative feeding experiences may impact clinical outcomes such as LOS, time to reach full feedings (oral or via tube), and safety of airway protection [23]. There is also disagreement as to whether preoperative feeding exposure may lead to better postoperative feeding outcomes [24, 25]. At our institution, patients under 12 months of age are automatically referred for a preoperative feeding/swallowing evaluation pending medical status and physiologic stability. Patients greater than 12 months are only referred pre/post-operatively if they have a history of or demonstrate signs of dysphagia. Preoperative feeding experiences range from pre-feeding support, including skin to skin/kangaroo care, oral stimulation via pacifier, tastes of breast milk for oral care and pre-feeding experiences, to breast and/or bottle feeding. Clinicians must work closely with the medical team and rely on highly skilled analyses to determine oral feeding readiness and safety of PO intake as standardized assessments and protocols are limited [9, 26]. In our cohort, of the 168 patients who had a preoperative clinical swallow evaluation, 51 (30.4%) had clinical concern for aspiration preoperatively and 42 (82.5%) of these patients were discharged on tube feeding postoperatively. This suggests that there is a high level of baseline dysphagia in patients with CHD even prior to CHD surgery. Conversely, of the 117 patients who had no signs of aspiration on their preoperative clinical swallow evaluation, 72 (61.5%) were ultimately discharged home postoperatively requiring tube feeding, indicating as expected that many patients develop dysphagia after surgical intervention for CHD. Therefore, the findings on preoperative swallowing assessment while helpful should be interpreted with caution in the overall feeding plan at discharge.

All infants less than 12 months of age with CHD at our institution are also referred for a postoperative feeding assessment. Our postoperative clinical feeding assessment often includes a secondary oral mechanism exam, evaluation of feeding readiness signs and physiological stability, subjective assessment of vocal quality and respiratory stability, and oral feeding/swallowing assessment via breast or bottle. Infants in the cohort that demonstrated concerns for aspiration on postoperative clinical swallowing evaluation, were then referred for an instrumental swallowing exam. Of the 350 patients who underwent a postoperative clinical swallowing evaluation, 221 (63.4%) demonstrated clinical concern for aspiration indicating need for VFSS. In our cohort, both preoperative and postoperative clinical concerns for dysphagia were significant for predicting the need for tube feeding upon discharge. This highlights that identifying dysphagia early in the clinical course can help guide the feeding plan and provide attendable caregiver expectations [19, 21, 27].

### Instrumental Swallowing Exam

Relying on clinical swallow evaluation alone, without use of an instrumental exam, may lead to missed diagnosis of dysphagia, aspiration and inaccurate caregiver expectations regarding oral feeding success [28]. VFSS is considered the gold standard and most widely used instrumental swallowing assessment for identifying dysphagia [29, 30]. Raulston et al., found that a high prevalence of silent aspiration exists in the post-surgical CHD patients. 73% of patients in their study of infants less than 12 months old who underwent VFSS after cardiac surgery demonstrated silent aspiration [31].

In our study, most patients who underwent a postoperative instrumental swallow study had mild-moderate dysphagia (median DOSS 4). A mild-moderate dysphagia indicates that one to two liquid consistencies are limited, often resulting in need for thickened liquids and/or tube feedings. Sixty-seven percent of patients had severe dysphagia that required tube feeding as their primary diet at discharge. Similarly, Karsch et al.[32] demonstrated that of 62 postoperative single ventricle patients they studied, 46% aspirated or were at risk for aspiration (indicated by laryngeal penetration). Tube feeding was required by 66% of their participants.

We also found that the longer the time between surgery and the first postoperative clinical swallow evaluation, the more likely a patient was to need tube feeding at discharge. We postulated that this delay was a surrogate marker for medical instability which impeded the initial postoperative feeding evaluation. Immediate postoperative concerns such as prolonged intubation, need for respiratory support or delayed sternal closure can contribute to delayed feeding initiation/evaluation. Post-extubation dysphagia is widely reported with a higher likelihood for patients who have extended periods of intubation [33, 34]. Similarly, Souza’s 2018 study found that there was a statistically significant correlation between intubation duration (> 24 h) and presence of dysphagia [5]. Hoffmeister et al.’s retrospective review of 372 patients noted dysphagia in 29% of patients and for every hour of intubation the odds increased by 1.7% [35]. A delay in postoperative feeding evaluation is associated with an increased need for tube feeding; this information can be helpful with determining a feeding plan and caregiver counseling.

### Primary and Supplemental Diet

Institutional practices on thickening liquids for infants vary widely and can be controversial [36]. Nearly half of our cohort (51.3% on tube feeding vs 36.8% not on tube feeding) required a diet utilizing thickened oral feeds. According to a systematic review by Gosa et al. in 2011, the evidence available supporting the use of thickened liquids in pediatric dysphagia management is from studies that are small, have non-experimental design, or report mixed results [37]. The thickening practices vary in our institution and range from the use of infant cereal to thicken formulas, Gel mix with low-risk infants on breast milk, purees and commercial thickeners such as Simply thick and Thick it for our older patients without risk of GI complications. In our cohort, patients with severe dysphagia (DOSS score of 1) or patients not appropriate for thickened feeds often were discharged with limited oral volumes of 5–10 ml Level 0 milk, 5-10 mL by bottle or pumped breast, to maintain oral skills despite documented aspiration risk on an instrumental exam.

### Single Ventricle Cardiac Diagnosis

Numerous previous studies have found that single ventricle cardiac disease is associated with feeding difficulties and severe dysphagia requiring tube feeding at discharge [22, 23, 38–40]. Several factors are thought to contribute to this including delayed oral feeding preoperatively, multiple medical and surgical interventions that occur during times of feeding development, as well as an increased risk of developing necrotizing enterocolitis in this population. The concern for necrotizing enterocolitis has led to controversy regarding the safety of feeding patients receiving prostaglandins while they await surgical palliation [22, 39]. There are national initiatives through NPC-QIC to initiate feeding orally preoperatively that has not yet been proven to impact the need for tube feeding at discharge [41].

Based on clinical findings single ventricle patients may undergo PDA stenting/pulmonary banding, aortopulmonary shunting, or the Norwood procedure. We found a higher rate of severe dysphagia requiring tube feeding at discharge in children who had a Norwood repair (Norwood *p* < 0.0005 (OR 10.6 CI 3.3–54.6) and Blalock-Thomas-Taussig shunt (*p* = 0.046 (OR 2.4 CI 0.95–6.7). There was also a higher rate of VFMI in those who had a Norwood (*p* = 0.009) and Blalock-Tausig shunt (*p* = 0.048) patients compared to patients that underwent PDA stenting or pulmonary banding. DOSS scores were not statistically different among the different surgical groups, but available instrumental swallow studies were limited in the PDA stent group.

### Vocal Fold Motion Impairment (VFMI)

Fifty-six percent of patients with VFMI in our cohort were discharged home on tube feedings. The literature is inconsistent regarding the association between VFMI and need for tube feeding [28]. According to Connelly et al.’s study on vocal cord paralysis and dysphagia after aortic arch reconstruction and Norwood procedure, dysphagia was prevalent with or without VFMI [42]. By contrast, the majority 53% of the 96 patients in Raulston et al.’s 2019 study of patients with CHD with normal vocal cord function postoperatively had aspiration on the instrumental exam [31]. At the time of this retrospective review a subjective voice assessment pre and postoperatively is included in the clinical swallow evaluation, however laryngoscopy performed by the otolaryngology team was only performed based on symptoms or aspiration noted on instrumental swallow evaluation. Due to the association of VFMI and severe dysphagia in our study data we advocate for universal postoperative laryngoscopy for vocal fold evaluation in all CHD patients undergoing procedures deemed to be high risk for recurrent laryngeal nerve injury. If VFMI is identified, that should be another indication for patients to undergo an instrumental evaluation of swallowing.

### Standardized Feeding Protocol for at Risk Populations

**Fig. 2:**
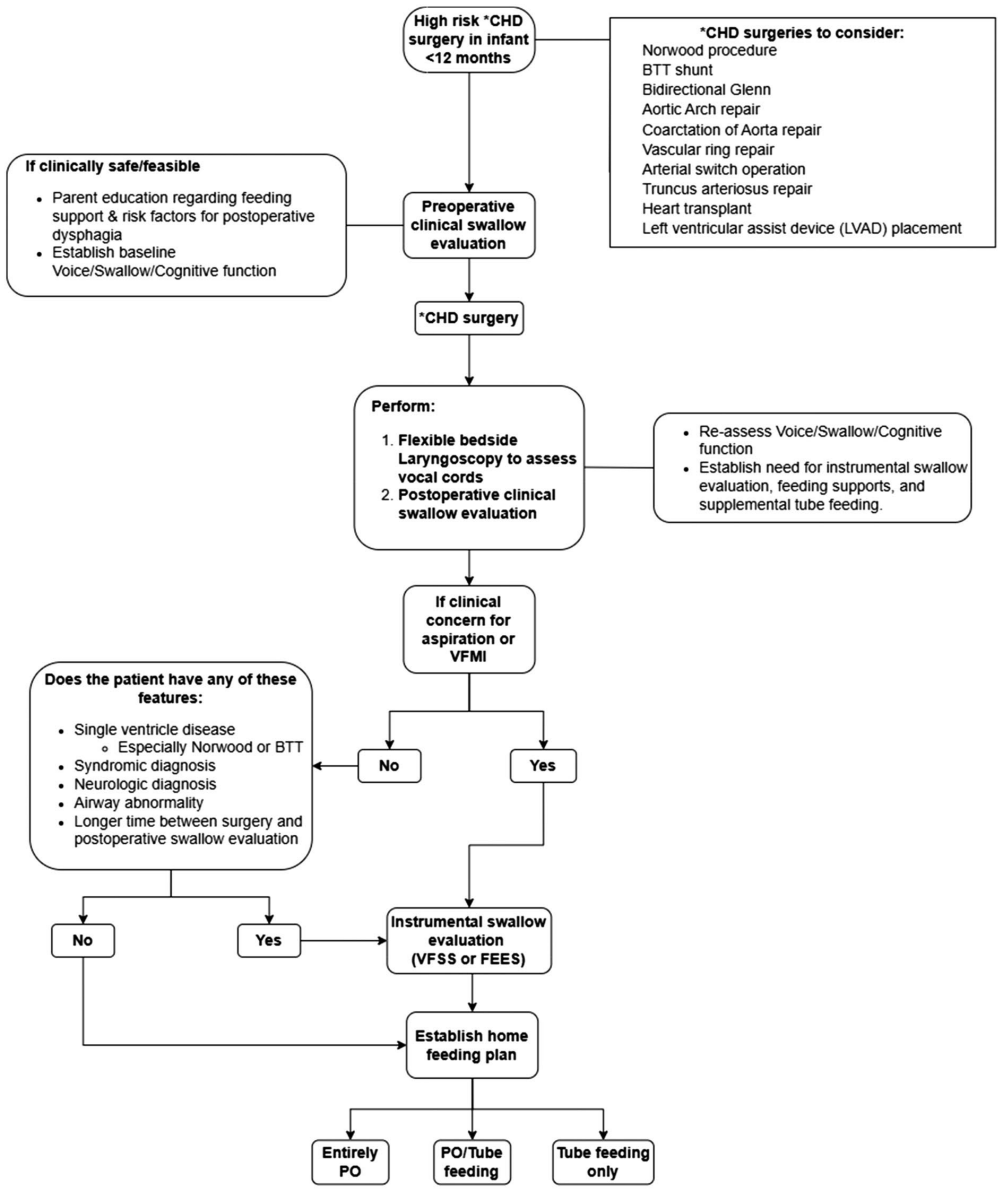
Proposed protocol for management of feeding in patients undergoing CHD surgery that is high risk for dysphagia and VFMI (as listed) at < 12 months of age. *CHD* Congenital heart disease, *BTT* Blalock-Thomas-Taussig, *VFMI* Vocal fold motion impairment, *PO* per oral

Based on these identified factors, we propose a clinical protocol to standardize feeding assessment and vocal fold evaluation in children less than 12 months undergoing CHD surgery with a high risk of postoperative dysphagia and VFMI (Fig. 2). A multidisciplinary team approach that includes Speech and Language Pathology, Nursing, Cardiology, Surgery, Nutrition, and Otolaryngology is essential in determining an effective feeding plan.

Specific attention and management efforts should be targeted to patients undergoing surgery at less than 12 months. In our cohort, 197 patients (63,5%) that underwent surgery at < 12 months of age compared to 35 patients (39.8%) that underwent surgery > 12 months of age required tube feeding at discharge (*p* < 0.005). In addition, patients with single ventricle cardiac disease, postoperative clinical signs of aspiration, a prolonged postoperative time to initiate feeding and those with VFMI should also be targeted as they are more likely to have feeding difficulties and need tube feeding at discharge. Completion of a pre- and post-operative clinical swallow evaluation, instrumental swallowing exam—VFSS and/or FEES and Otolaryngology consult with laryngoscopy would establish a safe and effective home/discharge feeding plan that supports oral feeding skills development and the necessity to gain weight and thrive.

### Limitations

The major limitation of this study is the retrospective single major medical center design, limiting the generalizability of results. There is an opportunity for greater collaboration across centers to help sort through the multifactorial nature of feeding difficulties in this population. Also due to the retrospective nature of the study instrumental swallow outcomes were not available in all patients. This is an issue with most studies on this topic and can make direct comparison of results difficult. Regression modeling in a retrospective study can also be challenging due to missing variables which can limit a model’s degrees of freedom. However, despite this we were able to attain a model with a c-statistic of 0.85 indicating a good reliability model. Most of the literature looks at infants and neonates < 12 months when investigating dysphagia in the CHD population our sample is a cross-sectional evaluation of all patients undergoing cardiac surgery that had a swallowing evaluation with speech therapy—even despite this the median age in our cohort was still 1.1 months due to automatic SLP evaluation orders. We favored this design as it would allow the different subgroups to serve as controls and the developed standard protocol be more reflective of daily clinical practice.

## Conclusion

Dysphagia is a common sequela after cardiac surgery for patients with CHD, with many patients having severe dysphagia requiring tube feeding at the time of discharge. We found several factors that are associated with the need for tube feeding at discharge and developed a protocolized, multidisciplinary approach to evaluate and manage dysphagia to establish and monitor oral feeding safety and optimize nutritional needs.

## Data Availability

No datasets were generated or analysed during the current study.
